# Rheology and Printability of Hydroxyapatite/Sodium Alginate Bioinks Added with Bovine or Fish Collagen Peptides

**DOI:** 10.3390/gels11030209

**Published:** 2025-03-15

**Authors:** Mario Milazzo, Roberta Rovelli, Claudio Ricci, Teresa Macchi, Giuseppe Gallone, Serena Danti

**Affiliations:** 1 Department of Civil and Industrial Engineering, University of Pisa, 56122 Pisa, Italy; claudio.ricci@unipi.it; 2 Consorzio Interuniversitario Nazionale per la Scienza e Tecnologia dei Materiali (INSTM), Via G. Giusti 9, 50121 Florence, Italy; teresa.macchi@phd.unipi.it; 3 The BioRobotics Institute, Sant’Anna School of Advanced Studies, Viale Rinaldo Piaggio, Pontedera, 56025 Tuscany, Italy; 4 PEGASO Doctoral School of Life Sciences, University of Siena, 53100 Siena, Italy; r.rovelli@student.unisi.it; 5 Department of Translational Research and of New Surgical and Medical Technologies, University of Pisa, Via Savi 10, 56126 Pisa, Italy

**Keywords:** 3D printing, hydrogel, rheology, biomaterials, bone scaffold

## Abstract

The high biocompatibility and the key role of collagen in bone extracellular matrix make it useful for tissue engineering. However, the high demand, costs, and challenges of extracting good-quality collagen have led to the use of collagen derivatives and search for non-human alternatives. This study investigates fish and bovine collagen peptides (Coll_f_ and Coll_b_, respectively) as sustainable sources for 3D-printed bone scaffolds by developing and characterizing peptide-incorporated alginate/hydroxyapatite-based bioinks. The chemical analysis revealed structural similarities between the peptides, while rheological tests showed a slightly higher viscosity of Coll_f_-based inks, which improved shape fidelity during the printing process. Upon oscillating rheological tests, both the Coll_f_ and Coll_b_-based ink formulations demonstrated a solid-like behavior at frequencies higher than 0.4 Hz, which is crucial for maintaining the printed structure integrity during extrusion. Although Coll_b_-based inks exhibited better pore printability, Coll_f_-based inks achieved superior resolution and geometry retention. Macro-porous structures printed from both inks showed good accuracy, with minimal shrinkage attributed to hydroxyapatite. Both the produced inks had a high gel fraction and swelling behavior, with Coll_b_-based outperforming Coll_f_-based inks. Finally, both ink formulations resulted to be cytocompatibile with human dermal fibroblasts. These findings position Coll_f_- and Coll_b_-based inks as promising alternatives for bone tissue scaffolds, offering a sustainable balance between performance and structural stability in 3D printing applications.

## 1. Introduction

Bone tissue, with its complex hierarchical structure and remarkable mechanical properties, allows the human body to retain its structural integrity and function. When significant bone loss occurs due to trauma, disease, or congenital defects, the regeneration of large bone defects becomes a clinical challenge. Aiming to repair critical-size defects, conventional treatments, including autografts, allografts, and synthetic bone substitutes, are still considered the gold standard [1]. However, these strategies are limited by the scarce donor supply, potential patient site morbidity, and often suboptimal long-term outcomes [2,3]. These constraints have led to extensive research into alternative solutions, particularly in the field of tissue engineering (TE).

Bone TE encompasses novel materials and strategies that promote bone regeneration by mimicking the structure and function of the native bone extracellular matrix (ECM) [4,5]. The ECM of natural bone is a dense and well-organized composite material made of organic and inorganic components, with collagen and hydroxyapatite (HAp) being the most representative ones in the two categories, which provide both mechanical strength and biological features for cell proliferation and differentiation [4,6,7]. To replicate such an intricate microenvironment, the design of biomimetic scaffolds that are supportive for cellular activity and tissue repair has become increasingly important in developing new bone substitutes. Ideally, these scaffolds should possess mechanical properties and degradation rates appropriate for bone tissue regrowth and be biocompatible with bone cells so as to support cellular adhesion, proliferation, and osteogenic differentiation [3,8].

Collagen, which is the most abundant component of the organic bone ECM, has been extensively studied for its suitability in scaffolding due to its inherent biocompatibility, capacity of being bioresorbed by the human body, and ability to support cellular events crucial for bone repair [4]. However, the increased employment of collagen in biomedical applications and the difficulty of collagen extraction and processing have pushed scientists to explore alternative sources beyond human tissues, such as animal and recombinant sources, and use collagen derivatives, which are easier to process [9]. Nowadays, commercially available animal-based collagenous biomaterials are usually derived from mammalian tissues. Bovine collagen and its derivatives, such as gelatin and collagen peptides, are widely used due to their availability and low cost [9,10]. However, the risk of immunogenicity, zoonotic disease transmission, and hypersensitivity reactions, along with the associated religious and/or ethical constrains have researchers questioning its use. Moreover, differences in the quality of collagen, which can be influenced by the age, health, and physiological conditions of the donor animal, raise concerns about the reproducibility and consistency of bovine collagen-based scaffolds [10,11,12].

From this perspective, collagen from marine organisms has emerged as an attractive alternative, with fish collagen offering biological features similar to mammalian collagen, lower ethical issues, and religious restrictions, and a relatively low immune response [13,14]. Moreover, every year, the fishing industry generates a huge amount of marine waste or low-value byproducts (i.e., skin, heads, fins, bones, and scales), making the use of fish collagen not only profitable and cost-effective but also environmentally beneficial [15].

However, fish collagen is not free from limitations, particularly its poor cell seeding efficiency and lower denaturation temperatures, which may compromise its mechanical resilience and lead to fast in vivo biodegradation [16]. To address these issues, composites scaffolds incorporating marine collagen have been designed for bone TE. For instance, Pallela et al. designed a composite collagen scaffold mimicking the natural bone ECM by combining marine collagen sourced from *Ircinia fusca* sponge with natural HAp and a chitosan matrix. The scaffold demonstrated enhanced porosity, thermal stability, and increased proliferation of human osteoblast-like cells (i.e., MG-63) [17]. Similarly, a composite sponge made of type II collagen from blue shark (i.e., *Prionace glauca*) cartilage combined with HAp and chitosan was developed for guided bone regeneration. This scaffold efficiently promoted the viability of osteoblast-like cells (i.e., hFOB12) and significantly increased alkaline phosphatase activity, thus suggesting the suitability of fish collagen as an alternative to mammalian collagen sources in bone TE applications [18].

With the advent of additive manufacturing techniques, and specifically three-dimensional (3D) bioprinting, scaffold design has significantly evolved toward enabling the creation of highly customized structures that can closely mimic the native bone microarchitecture. These technologies allow the obtainment of a tailored scaffold shape, porosity, and pore interconnectivity, which are critical parameters for successful bone regeneration [19,20,21], thus resulting in a versatile approach to address the complex architectures and topologies with respect to traditional methods, like solvent casting/particulate leaching, freeze drying, and gas foaming [22]. Therefore, collagen-based hydrogels, which are frequently used as bioinks in 3D bioprinting, are favored for their similarity to the natural ECM, biocompatibility, and tunable mechanical properties, creating an optimal environment for cellular growth [22,23,24].

Even though fish collagen can offer a new alternative in bone TE [25,26,27,28], standalone collagen solutions face challenges in printability due to their low viscosity and mechanical strength, leading to the inclusion of supportive polymers to enhance their printability. In particular, sodium alginate (SA), a polysaccharide derived from brown algae, has been deeply investigated in bioink systems due to its low cost, biocompatibility, favorable rheological properties, and fast gelation process via crosslinking with bivalent ions [29,30]. For instance, Diogo et al. developed a printable bioink by blending mineralized blue shark (i.e., *Prionace glauca*) collagen with SA. The resulting hydrogel formulation demonstrated good printability and mechanical properties, which effectively supported fibroblast viability and proliferation, highlighting its potential for engineering hard tissues [31]. Similarly, Govindharaj et al. incorporated eel collagen into a SA-based hydrogel to fabricate scaffolds using 3D printing technology. The hydrogel-based ink showed excellent printability and cytocompatibility, along with superior cellular metabolic activity and proliferation [26]. Despite these promising features, previous investigations offered a comparison between mammalian and fish collagens, mainly from a biological perspective. Collagen derivatives are water-soluble, lower-molecular weight, and easier to process than collagen biomolecules but still retain the biochemical fingerprints of the native collagen, which supports important cellular events, like adhesion and proliferation. Fish collagen tends to have lower hydroxyproline and glycine levels and reduced thermal stability than mammalian collagen. Despite both being typically Type I collagen, fish collagen has fewer intramolecular crosslinks, enhancing its solubility and bioavailability, and when hydrolyzed into peptides, its lower molecular weight and reduced amino acid content improve absorption and solubility compared to mammalian versions.

In our study, we addressed the development of a multi-component bioink for 3D printing made of SA and HAp and incorporating collagen peptides from two different animal sources, namely, Coll_f_ and Coll_b_, for bone TE applications. Manufacturing features as well as the physical and biological characterization were performed for the two bioink types.

Overall, understanding how collagen from different sources could be exploited for scaffold design, including printability and cell interactions, is crucial for optimizing 3D bioprinting strategies aimed at fabricating biomimetic tissue replacements without the need for human collagen.

## 2. Results and Discussion

### 2.1. FTIR Analysis of Collagen Peptides

**Figure 1:**
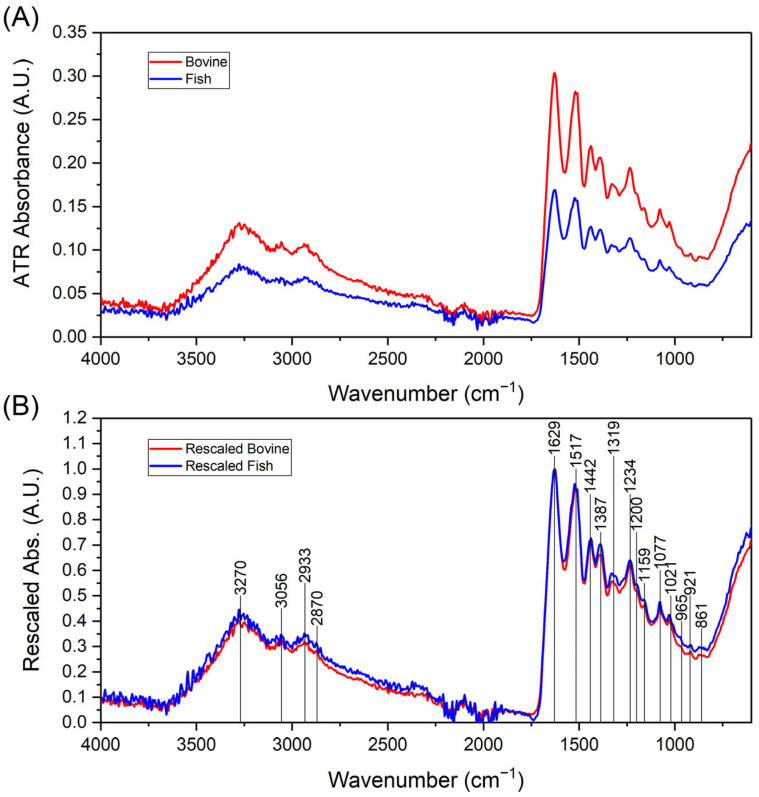
(**A**) FTIR spectra of the Coll_b_ and Coll_f_ powder samples. (**B**) FTIR spectra after rescaling (see text), together with the identification of the relevant absorption bands.

Coll_f_ and Coll_b_ were investigated via an FTIR analysis to assess possible major differences in their chemical compositions and structures. By comparing the spectra obtained for the two collagen peptides, no appreciable differences in the chemical structure could be assessed. As reported in Figure 1, both samples presented main peaks at wavelengths equal to 3250 cm^−1^, between 1500 and 1600 cm^−1^, and at 500 cm^−1^. Figure 1 reports the IR spectra collected from both bovine and fish collagen samples. In particular, Figure 1A shows the raw absorption data acquired in ATR mode for both type of samples. In all cases, the typical spectrum evidences the presence of two main regions of relevant absorbances, respectively, located in the 700–1700 cm^−1^ and 2800–3500 cm^−1^ wavenumber intervals, which are recognizable as the characteristic pattern of IR bands from various types of collagen [32,33].

Bovine and fish samples exhibited similar IR absorption patterns and their spectra can almost be superimposed when, after the subtraction of the background, they were rescaled through normalization to the intensity of the common largest peak at 1629 cm^−1^, as shown in Figure 1B. It is then evident that the two spectra share the same positions of the recognizable absorption bands and that there are only slight differences in the relative absorbances that correspond to some of such bands that can reasonably be attributed to the differences in the composition between the two samples in terms of the various amino acids, as declared in the technical datasheets from the vendor. Figure 1B also reports the positions of those absorption peaks that could be unambiguously located through an analysis of the second derivatives of the spectra. Thanks to some previous works that studied the FTIR responses of various types of collagens [32,33], it is straightforward to assign the IR absorption peaks, as detailed in Table 1, and recognize the occurrence of the chemical details typical of polypeptides in both samples.

### 2.2. Rheological Characterization

**Figure 2:**
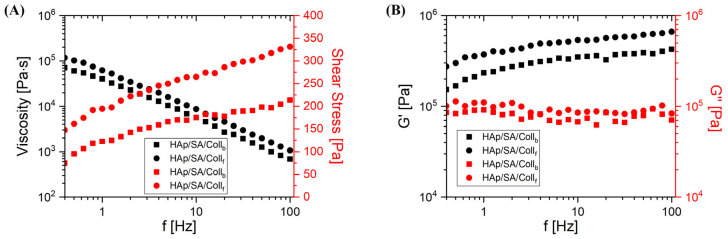
Rheological characterization of the solutions as a function of the frequency from 0.4 to 100 Hz. (**A**) Viscosity and shear stress; (**B**) storage (*G′*) and loss (*G′′*) moduli.

Figure 2 reports the rheological characterization of uncrosslinked bioinks containing either Coll_f_ or Coll_b_.

Both solutions presented an unstable behavior when tested at frequencies up to 0.3 Hz: the measured values were affected by random scattering (typically, sudden drops of values) and were unreliable, so that even the same instrumental apparatus automatically tagged those data with one or more error status codes soon after their acquisition. Such indications of possible issues and loss of significance are the reasons why the data at frequencies below 0.4 Hz are not presented here. By considering the flow curves in Figure 2A, a rheological behavior emerged for both samples that is typical of non-Newtonian and shear thinning liquids, as suggested by the significant decrease in the viscosity observed as the frequency increases, in parallel with a clear increase in the shear stress. However, the fish-based solution presented slightly higher values of the viscosity than those of the bovine one. An interesting scenario also arose from Figure 2B, where the storage (G′) and loss (G′′) moduli are presented as a function of the frequency. The trends observed for G′ and G” indicated that our systems behaved as viscoelastic liquids. In fact, both samples exhibited a storage modulus (G′) that moderately increased with frequency and a loss modulus (G”) that, although it was almost independent of the frequency, was comparable in magnitude to G′. Nevertheless, for both samples, G′ > G′′ occurred over the whole frequency range, which is typical of a solid-like behavior. This suggests that such systems can be regarded as being structured to some extent, either because HAp nanoparticles associated, because SA acquired a gelled state (hydrogel-like), or because of both the mentioned factors. In this view, interpreting the data as the result of a tendency of our solutions to acquire a certain level of structuring could also explain the issues reported at the beginning of this paragraph about the measurements at very low frequencies.

In fact, given the composition of these systems, it can be reasonable to expect that, soon after the formulation of the solutions, some structured domains may readily start to form therein. Such structures might either be aggregates of HAp particles, physical crosslinks from the alginate hydrogel, or both. At the beginning of each test, that is, at the initial timepoints of each frequency sweep and when the shear rates are yet very low, those structures that are still too weakly bonded will be progressively disaggregated over time by the action of the oscillatory shear strain, and this will result in sudden variations in the rheological response of the specimen under test, very likely showing up in the form of random drops in viscosity. Such an uncontrollable behavior will continue until more stable phase conditions and response regime are attained, as it seems to occur here at successive stages of the sweep above 0.4 Hz.

### 2.3. Printability Assessment

**Figure 3:**
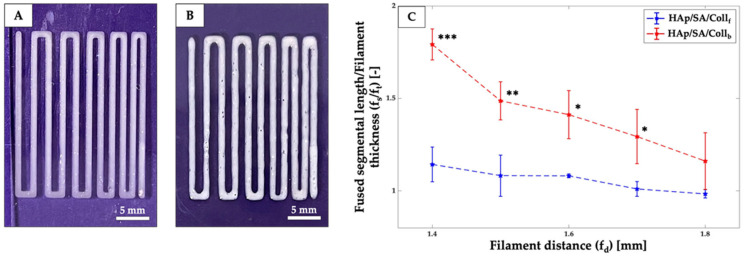
Filament fusion test results for (**A**) the HAp/SA/Coll_f_ and (**B**) HAp/SA/Coll_b_ inks. (**C**) Plot illustrating the relationship between f_s_/f_t_ and f_d_ for the HAp/SA/Coll_f_ and HAp/SA/Coll_b_ inks. *** *p* ≤ 0.001, ** *p* ≤ 0.01, and * *p* ≤ 0.05.

The bioinks were obtained by mixing SA and HAp with collagen peptides (either Coll_f_ or Coll_b_) in water, leading to composite “HAp/SA/Coll_f/b_” inks with a 5.5/10/10 weight ratio between the three components. The filament fusion pattern was printed with both HAp/SA/Coll_f_ and HAp/SA/Coll_b_ inks (Figure 3). A slight tendency for adjacent filaments to fuse was observed, becoming more pronounced as the filament gap distance decreased. Specifically, as the filament distance (f_d_) decreased, an increase in filament length (f_s_) was observed for both HAp/SA/Coll-based inks. At the minimum f_d_ (i.e., 1.4 mm), the HAp/SA/Coll_f_ ink exhibited a significantly lower f_s_ value (*p* ≤ 0.001) compared to HAp/SA/Coll_b_ ink, indicating a superior printing resolution and the ability to print finer structures.

**Figure 4:**
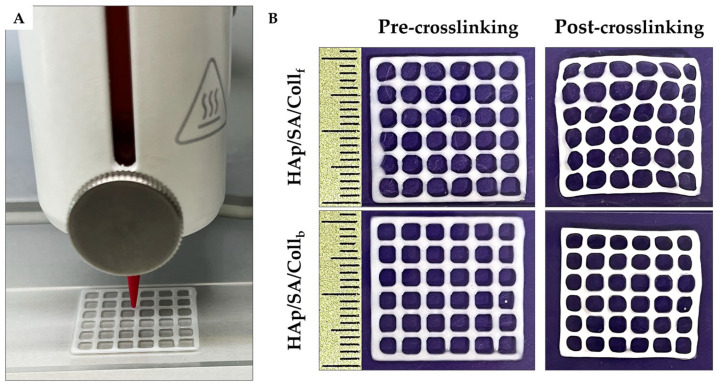
(**A**) Representative image of the 3D printing process for 20 × 20 × 0.6 mm square grids using Hap/SA/Coll-based inks. (**B**) Printability evaluation of 20 × 20 × 0.6 mm^3^ square grids of HAp/SA/Coll_f_ and HAp/SA/Coll_b_ grids in both uncrosslinked and crosslinked states.

The two inks were further tested by printing custom-designed porous structures (i.e., 20 × 20 × 0.6 mm^3^ square grids), which were further crosslinked using a 1% (*w*/*v*) calcium chloride aqueous solution. The outcomes of 3D printing for Coll_f_- and Coll_b_-based composite bioinks are shown in Figure 4. The shape fidelity of these structures was evaluated by measuring the printability (*P_r_*) and diffusion rate (*D_r_*) parameters, as summarized in Table 2.

Both HAp/SA/Coll-based inks demonstrated good printability (i.e., *P_r_* values close to 1.0). Specifically, the HAp/SA/Coll_b_ ink exhibited significantly superior pore printability compared to the HAp/SA/Coll_f_ ink, showing *P_r_* values of 0.85 ± 0.01 compared to 0.82 ± 0.01 (*p* ≤ 0.001). *P_r_* values lower than 1.0 indicated that the inks were slightly under-gelled, leading to scaffold pores that appeared more rounded than squared.

Hence, the *D_r_* value was measured to evaluate the pore closure effect caused by filament spreading during printing. The HAp/SA/Coll_f_ ink showed a significantly smaller diffusion rate than the HAp/SA/Coll_b_ ink, with *D_r_* values of 39.51 ± 3.41 and 43.72 ± 3.39, respectively (*p* ≤ 0.01), suggesting an improved ability to maintain the designed structure. However, the printed shape was better preserved after crosslinking with the HAp/SA/Coll_b_ ink compared to the HAp/SA/Coll_f_ ink, as indicated by the smaller shrinkage (*S*) values of 12.88 ± 0.85% and 13.62 ± 1.41%, respectively (Table 2).

### 2.4. Gel Fraction Analysis

The gel fraction analysis was carried out on both the HAp/SA/Coll_f_ and HAp/SA/Coll_b_ inks to assess the percentage of crosslinks within the hydrogel network (*n* = 3). The gel fraction percentage (*Gf%*) for the two collagen peptides-based formulations is displayed in Table 3. By performing weight loss measurements of the dried crosslinked bioink samples as prepared and after immersion in water for 24 h, the Gf% for HAp/SA/Coll_f_ was 88.2 ± 10.1%, while that for HAp/SA/Coll_b_ was 90.7 ± 6.7%. These findings were further investigated by assaying the quantity of released collagen peptides from the hydrogel networks using the Bicinchoninic Acid (BCA) test. No detectable release of HAp was either visually or microscopically observed, suggesting its stable incorporation within the hydrogel matrix. By detecting the amount of released collagen peptides, which was slightly but not significantly higher in Coll_b_-based inks, it was also possible to estimate the fraction of crosslinked alginate, namely, the alginate that remained inside the hydrogel matrix, whose amount resulted to be less consistent in Coll_f_-based inks with respect to Coll_b_-based counterparts (Table 3).

### 2.5. Swelling Test

**Figure 5:**
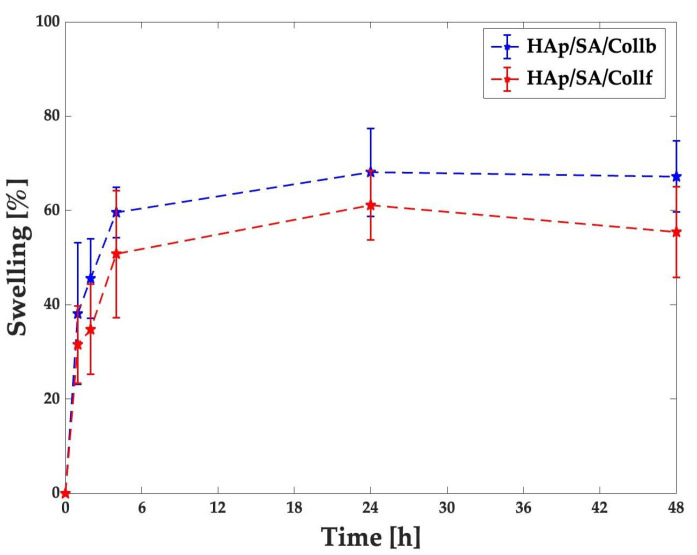
Swelling behavior of the crosslinked HAp/SA/Coll_f_ and HAp/SA/Coll_b_ inks for up to 48 h in PBS at 37 °C.

The swelling behavior of both the HAp/SA/Coll_f_ and HAp/SA/Coll_b_ inks was investigated after crosslinking by monitoring their weight variations over time under simulated biological conditions, i.e., phosphate-buffered saline solution (PBS) at 37 °C (Figure 5). Both hydrogel formulations initially appeared to absorb a high amount of PBS, followed by a less pronounced increase until reaching a well-developed swollen form in 4 h. The maximum swelling was detected at 24 h, at which the swelling percentage was 61 ± 7% for HAp/SA/Coll_f_ and 68 ± 9% for HAp/SA/Coll_b_ samples. For both inks, a slight reduction in the average swelling rate, without statistical significance, was observed after 24 h, indicating the establishment of equilibrium, in which osmotic forces counterbalanced the elastic forces of polymer network, thereby limiting further swelling.

### 2.6. Cytocompatibility Evaluation

The cytocompatibility of the produced bioinks was investigated by performing an indirect cytotoxicity test using the resazurin metabolic activity assay and human dermal fibroblasts (HDFs). Both scaffold types supported the normal metabolic activity in HDFs, with no statistically significant differences among the two tested materials. The results indicated HDF metabolic activities of 88.8 ± 4.4% and 91.75 ± 3.9% after being exposed for 24 h to culture media conditioned with HAp/SA/Collf and for HAp/SA/Collb, respectively.

### 2.7. Discussion

The search for effective alternatives to existing bone grafting approaches has driven the development of 3D engineered bone tissue analogues. In this context, collagen has been widely explored for scaffolding due to its inherent biocompatibility and its role as a key component of the native bone ECM. However, the increasing demand for collagen, along with the risks associated with mammalian-derived sources, has led researchers to explore collagen from marine organisms as a promising alternative. Aiming to assess the potential of marine collagen for bone TE, in this study, we compared collagen peptides from fish and bovine sources to finally prepare HAp/SA/Coll bioinks for 3D printed bone substitutes.

Before the fabrication of the inks, an FTIR test showed that the two sources did not present major differences in their chemical composition, with absorbance peaks corresponding to those observed in human collagen [32].

The rheological characterization performed on the inks revealed an interesting scenario in which the viscosity and the associated shear stress presented opposite behaviors, with a marked decrease in the intrinsic properties of the compounds at increasing frequencies. The viscosity is, indeed, a critical physiochemical property influencing the performance of 3D printable inks. The Coll_f_-based ink possessed a slightly higher viscosity than the bovine compound across frequencies above 0.4 Hz, which could offer advantages in maintaining the shape fidelity of the 3D-printed structures. The differences in viscosity between native collagen and its derivatives, such as collagen peptides, are pivotal in this context. Native collagen exhibits higher viscosity due to the strong electrostatic repulsion among its molecular chains, even in low-concentration solutions. Bonnie Sun et al. showed that hydrolyzed collagen, with its low molecular weight and short chain segments, maintained a very low viscosity, regardless of the concentration [34]. Marine collagen-based bioinks displayed a lower viscosity than their mammalian counterparts, in accordance with previous investigations related to highly stranded collagen molecules [35].

Independent of the differences in the viscous behavior with respect to previous research, which were mainly due to the diverse compositions, in our study, both HAp/SA/Coll formulations were easily extruded via 3D printing into stable, continuous filaments using the same printing parameters, although a slightly higher extrusion pressure range was required for HAp/SA/Coll_b_ compared to HAp/SA/Coll_f_ (i.e., 13–16 kPa and 12–15 kPa, respectively). Additionally, in both formulations, the successful extrusion and stable filament formation were further sustained by their solid-like behavior (i.e., *G′* > *G′′*), which is essential for ensuring the structural integrity of the filament during and immediately after its deposition. To show the beneficial employment of HAp in 3D printing applications, Iglesias-Mejuto et al. reported that the inclusion of micro-sized HAp powder into a SA-based ink at concentrations up to 24 w% preserved the stand geometry while improving the structural stability and bioactivity of 3D-printed scaffolds [36]. Thus, combining collagen peptides with a HAp/SA-based hydrogel makes it possible to increase the advantageous use of collagenous derivatives without compromising the ink printability.

The concept of ink printability is not unequivocally defined and lacks an accepted, standardized method for its assessment. The printability window is often predicted by the ink’s rheological properties, such as viscosity, shear-thinning, and recovery behaviors, which overall help to establish the optimal printing conditions. In particular, following the results achieved in this work, the correct printability is supported by the solid-like behavior shown by both inks across frequencies. However, while these properties provide valuable insights for ink optimization, they are still insufficient to determine printability.

A comprehensive analysis of ink shape fidelity after printing is thus essential to assess the physical deformation of the printed strands and degree of similarity to the intended computer-aided design [37]. In this study, the printability of HAp/SA/Coll_f_ and HAp/SA/Coll_b_ inks was assessed through a filament fusion test, along with *P_r_*, *D_r_* and *S* factors. Both HAp/SA/Coll-based inks demonstrated good printing accuracy, with filament fusion patterns closely resembling the intended design. A negligible tendency for adjacent filaments to fuse was noted, becoming slightly more pronounced as the filament gap distance decreased. At the minimal f_d_ of 1.4 mm, the HAp/SA/Coll_f_ ink showed a significantly lower f_s_ compared to that of the HAp/SA/Coll_b_ ink, indicating a superior printing resolution and the ability to create fine structures, which are critical for developing scaffolds that closely mimic the microarchitecture of bone tissue.

The ink printability was further tested by printing custom-designed macro-porous structures (i.e., 20 × 20 × 0.6 mm^3^ square grids). Regardless, for the HAp/SA/Coll-based formulations, the 3D-printed grids exhibited a square pore shape and *P_r_* values near 1.0, indicating an ideal printability feature. Specifically, the HAp/SA/Coll_b_ ink showed a slightly superior pore printability (*P_r_* = 0.85 ± 0.01) compared to that of the HAp/SA/Coll_f_ ink (*P_r_* = 0.82 ± 0.01), thus suggesting that bovine collagen may confer better structural integrity immediately after printing. Notably, these *P_r_* values aligned with data reported for SA-based inks at similar concentrations (i.e., 4–6 w%), further supporting the reliability of the current formulations [38]. However, a higher diffusion rate was recorded for the HAp/SA/Coll_b_ ink (*D_r_* = 43.72 ± 3.39), indicating more significant pore closure and filament spreading compared to HAp/SA/Coll_f_ (*D_r_* = 39.51 ± 3.41). The lower *D_r_* value of the Coll_f_-based ink with respect to that of the Coll_b_-based ink, consistent with the fusion test results, highlighted its ability to maintain the printed geometry more effectively, which could be a crucial advantage when designing scaffolds that require precise architectural features for guiding cell proliferation and tissue integration.

To assess the inks’ capability of preserving the integrity of 3D-printed structures, the HAp/SA/Coll-based 3D-printed grids underwent crosslinking, and the shrinkage parameter was evaluated. Both formulations exhibited minimal *S*, with the Coll_b_-based ink offering a slightly better post-processing stability compared to its Coll_f_-based counterpart (i.e., 12.88 ± 0.85% and 13.62 ± 1.41%, respectively), making the Coll_b_-based ink more effective at preserving the scaffold dimensions after printing. Notably, both hydrogel formulations demonstrated superior structural integrity compared to pure alginate-based inks, potentially due to the inclusion of HAp particles [38]. Indeed, HAp in micro/nanosize formulations is known to promote gelation, as the slow dissolution of HAp can lead to a more gradual and sustained release of calcium ions; it also can improve the mechanical properties, allowing for extrusion-based 3D printing of stable filaments that stack effectively when combined with SA [36].

The structural stability of Coll_f_- and Coll_b_-based 3D-printed hydrogels was further investigated through a gel fraction analysis, providing insights into the effectiveness of crosslinking within the polymeric network. HAp/SA/Coll_f_ exhibited a higher gel fraction (i.e., 90.7 ± 6.7%) than HAp/SA/Coll_b_ (i.e., 88.2 ± 10.1%), thus suggesting a potentially tighter crosslinked polymeric network, which likely contributed to better shape retention and lower material release over time. Notably, no detectable release of HAp was observed from both Coll-based formulations, which confirmed its stable incorporation within both Coll_f_- and Coll_b_-based hydrogel matrices. This is particularly relevant for bone TE, as the HAp retention ensures mechanical support and long-term bioactivity. Consistently, Benedini et al. confirmed that ceramic additives like HAp are essential for improving scaffold performance and long-term stability, thereby supporting bone regeneration strategies [39]. By evaluating the amount of alginate that remained within the matrix, which could be considered as crosslinked alginate, we observed outcomes generally consistent with those derived from the gel fraction analysis, where the alginate weight fraction was higher in Coll_b_-based bioinks with respect to Coll_f_-based bioinks. Interestingly, on average, less alginate (i.e., 67.9%) and more widely distributed results (±29.2%) were detected in Coll_f_-based bioinks compared with those found in Coll_b_-based bioinks (91.4 ± 11.0%), which were not a consequence of the released collagen peptides, namely, 17.1 ± 5.2% in Coll_f_-based bioinks versus 23.8 ± 6.4% in Coll_b_-based bioinks. These findings may indicate a gel-stabilizing role of HAp, which is particularly relevant in Coll_f_-based bioinks. Overall, HAp can indirectly contribute to SA crosslinking in the presence of collagen peptides by slowly releasing calcium ions under favorable conditions and by enhancing the overall properties of the composite hydrogels [40].

The density of formed crosslinks influences the swelling capacity of the hydrogels by allowing or restricting water uptake within the polymer network. HAp/SA/Coll_f_ showed a slightly lower, but not statistically significant, swelling degree than that of HAp/SA/Coll_b_, aligning with its greater structural stability. Regardless of the collagen source, both collagen-based formulations reached swelling equilibrium in 4 h, with a maximum swelling degree at 24 h (i.e., 61 ± 7% and 68 ± 9% for HAp/SA/Coll_f_ and HAp/SA/Coll_b_, respectively). These values fall within the range reported for other 3D-printed natural polymer-based hydrogels reinforced with HAp [41], further supporting the importance of a well-regulated swelling behavior for bone TE, in which it promotes cell infiltration into the hydrogel and maximizes cell growth within the structure [42].

Finally, the cytocompatibility of both Coll-based bioinks was confirmed using the indirect cytotoxicity test, with no significant difference detected in the metabolic activity of HDFs after exposure to the compounds released by the materials. These findings reinforced the suitability of Coll_f_- and Collb-based formulations for various biomedical applications, including bone TE. In particular, the use of fish-derived collagen seems to be promising, even if its wider use hinges on improving the extraction and purification techniques to guarantee batch-to-batch uniformity. However, ongoing advancements in processing marine collagen from waste are expected to increase its commercial viability and make it a promising option for applications in regenerative medicine.

## 3. Conclusions

This study aimed to systematically compare the printability of Coll_f_- and Coll_b_-based inks for 3D-printed scaffolds for bone TE. The developed composite bioinks included SA for its gelling capacity and HAp to improve biological signaling and mechanical strength, along with collagen peptides from either a fish or bovine source to better mimic bone ECM features. Both Coll formulations showed favorable rheological and printability properties, together with post-printing structural integrity, due to the efficient gelling capacity of the composite bioinks, with the Coll_f_-based ink exhibiting superior shape fidelity and less filament spreading, which are essential requirements for accurate scaffold 3D printing. These findings establish fish collagen as an appealing alternative to mammalian-derived sources for bone TE. Overall, fish collagen and its derivatives can offer benefits in sustainability and biocompatibility for biomedical applications. Potential scalability issues are mainly due to the availability of large amounts of animal collagen with homogenous physico-chemical properties that can lead to reproducible results.

## 4. Materials and Methods

### 4.1. Materials

HAp in the form of a powder (average diameter less than 1 µm, molecular weight equal to 502.31 g/mol) was purchased from Acros Organics (Fisher Scientific, Hampton, NH, USA). Low-viscosity SA in powder form (average M_w_ = 427 kDa, code A1112) was purchased from Sigma–Aldrich (Milan, Italy). Collagen peptides from fish and bovine sources (average M_w_ = 2 kDa), in the form of a powder, were supplied by Lapi Gelatine S.p.A. (Empoli, FI, Italy). Dulbecco’s modified Eagle medium (DMEM), penicillin/streptomycin (PEN-STREP), L-glutamine, Dulbecco’s PBS (DPBS), and resazurin were bought from Merck (Darmstadt, Germany). Fetal bovine serum was purchased from GIBCO (Waltham, MA, USA), while Diflucan and trypsin EDTA were acquired from EuroClone S.p.A. (Milan, Italy). BCA Protein Assay Kit 23227 was purchased from Pierce (via Thermo Fisher Scientific, Waltham, MA, USA)

### 4.2. Fourier Transform Infrared Spectroscopy

FTIR spectroscopy was employed to investigate potential chemical differences between Coll_b_ and Coll_f_ samples in the form of powders. The samples were first desiccated at 40 °C for 24 h. The FTIR spectra were recorded in Attenuated Total Reflectance (ATR) mode with an Agilent Cary 630 FTIR spectrometer (Agilent Technologies Italia S.p.A., Cernusco sul Naviglio MI, Italy) by performing 16 scans in the 4000–500 cm^−1^ wavenumber region at a scanning resolution of 4 cm^−1^.

### 4.3. Preparation of HAp/SA/Coll Inks

The preparation of HAp/SA/Coll-based inks involved HAp and SA, along with Coll_f_ or Coll_b_, to produce two distinct inks, namely, HAp/SA/Coll_f_ and HAp/SA/Coll_b_. First, 10% (*w*/*v*) of the respective collagen peptides (either Coll_f_ or Coll_b_) was dissolved in distilled water (dH_2_O) at 60 °C with stirring for 30 min. Subsequently, HAp was added to the Coll solution at a concentration of 10% (*w*/*v*), ensuring proper dispersion. Immediately afterward, SA was incorporated in the same solution at a concentration of 5.5% (*w*/*v*). The mixture was kept at 60 °C under continuous stirring for at least 1 h, and then was gently stirred overnight at room temperature (RT) to ensure homogeneity before usage.

### 4.4. Rheological Characterization of the Inks

Rheological characterization of the inks was performed using an MCR 92 Rheometer (Anton Paar Italia, Rivoli, TO, Italy) equipped with a 25 mm parallel-plate measuring geometry sample cell. To avoid friction between the blend and the experimental equipment, we set a 50 µm fixed gap between the two steel plates. While reaching the correct gap distance, the excess material was carefully removed using a dedicated tool.

Preliminary tests on the samples were performed to evaluate the linear–elastic interval for both the investigated compositions. Thereafter, effective measurements were carried out to estimate the main rheological parameters, namely, the amplitude of the viscosity (*η*), the storage (*G′*) and loss (*G′′*) shear moduli, and the evolution of the shear stress (*τ*) as a function of the input frequencies (*f*), set between 0.05 and 100 Hz (*n* = 5).

### 4.5. The 3D Printing of Inks

A BioX 3D printer (Cellink, Gothenburg, Sweden) was used to print the HAp/SA/Coll-based inks. The inks were loaded into 3 mL cartridges (Cellink, Sweden) equipped with 27 G conical nozzles and dispensed pneumatically onto a movable plane using glass slides as printing substrates. The 3D-printed geometries were custom-designed in Solidworks^®^ 2024 and translated into G-Code via PrusaSlicer software (Version 2.5.0). Several tests were carried out to identify the optimal operating parameters for the successful printing of the inks. For this purpose, the extrusion pressure, deposition speed, z-offset, printhead temperature (T_t_), and print bed temperature (T_p_) were adjusted to determine the most effective combinations. After printing, the 3D constructs were maintained in a 1% (*w*/*v*) CaCl_2_ solution for 10 min to achieve physical crosslinking. Detailed information regarding the optimal printing parameters for both inks is provided in Table 4.

### 4.6. Filament Fusion Test

**Figure 6:**
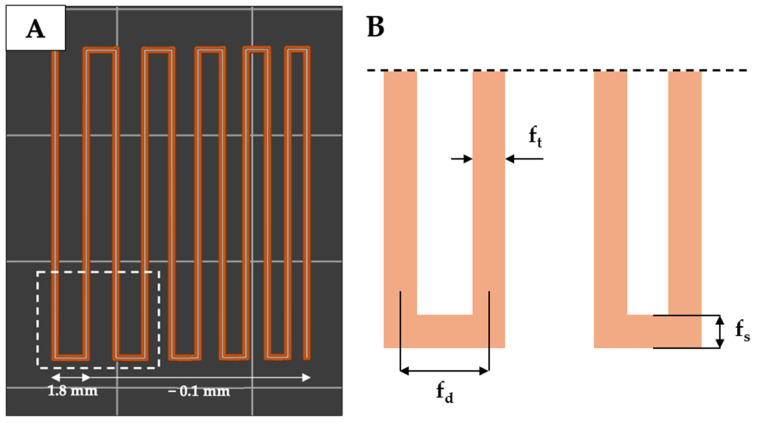
Filament fusion test. (**A**) Schematic illustration of the meandering pattern with decreasing filament distances for the filament fusion test. (**B**) Measurement definition for the meandering pattern: filament distance (f_d_), filament thickness (f_t_), and filament length (f_s_).

To evaluate the printing accuracy and shape fidelity of the inks, a filament fusion test was conducted based on the work of Ribeiro et al. [36]. This test involves printing a single-layer meandering pattern made up of parallel strands with progressively decreasing spacing (i.e., from 1.8 mm to 1.4 mm), as shown in Figure 6A. The fused segment length (f_s_) and filament thickness (f_t_) were measured using ImageJ software (Version 1.54p) to assess the tendency of filament fusion and printing resolution. The filament distance (f_d_) was obtained directly from the designed pattern (Figure 6B). The ratio of f_s_ to f_t_ (f_s_/f_t_) was calculated to account for the influence of the filament thickness.

### 4.7. Semi-Quantification of Printability

To quantify pore closure and shape fidelity for the two inks, *P_r_* and *D_r_* were assessed. Specifically, square grids measuring 20 × 20 × 0.6 mm^3^ were 3D printed for this purpose. The *P_r_* value is defined by comparing the circularity of a square (π/4) with the resulting pore, as in Equation (1):

(1) $$P_{r}=\frac{L^{2}}{16A}$$

where *L* and *A* are the perimeter and area of the pore, respectively. A perfect square pore has a *P_r_* value of 1.0, while *P_r_* < 1 indicates under-gelled material and *P_r_* > 1 suggests over-gelled material.

The *D_r_* value relates to the area reduction of the actual pore size compared to the theoretical pore size, as defined in Equation (2):

(2) $$D_{r}=\frac{A_{t}-A_{a}}{A_{t}}$$

where *A_t_* and *A_a_* are the theoretical and actual areas of the pore, respectively. Ideally, *A_a_* equals *A_t_*, resulting in a *D_r_* of 0%, which means no material spreading and stable pores according to the pre-designed 3D model.

To evaluate the influence of crosslinking on shape fidelity and accuracy, the shrinkage value (*S*) was calculated as shown in Equation (3):

(3) $$\underline{S}=\frac{{\underline{S}}_{x}+{\underline{S}}_{y}}{2}=\frac{\left(\frac{{\underline{L}}_{bx}-{\underline{L}}_{ax}}{{\underline{L}}_{bx}}\times 100\right)+\left(\frac{{\underline{L}}_{by}-{\underline{L}}_{ay}}{{\underline{L}}_{by}}\times 100\right)}{2}$$

where ${\underline{S}}_{x}$ and ${\underline{S}}_{y}$ are the arithmetic means of the shrinkage in the *x*-/*y*-directions, ${\underline{L}}_{bx}$ and ${\underline{L}}_{by}$ are the average side lengths in the respective directions before crosslinking, and ${\underline{L}}_{ax}$ and ${\underline{L}}_{ay}$ are the average side lengths after crosslinking. Smaller shrinkage values indicate a better capacity of the ink to preserve the printed shape, while larger values suggest greater contraction and potential distortion. All geometric parameters were measured using ImageJ software.

### 4.8. Gel Fraction Analysis

The crosslinking degree of the hydrogel network was evaluated by measuring the gel fraction for the two inks. Square samples (10 × 10 × 1 mm^3^, *n* = 3) were 3D printed for each collagen-based formulation and crosslinked by immersion in 1 mL of a 1% (*w*/*v*) CaCl_2_ solution for 10 min. Afterward, the samples were soaked in dH_2_O, dried in an oven at 50 °C for 1 h, and weighed to obtain the initial dry weight (W_0_). The samples were placed in a 24-well plate, immersed in 1 mL of dH_2_O at RT for 24 h to remove soluble components, then dried at 50 °C, and weighed again (W_f_). The gel fraction percentage (*Gf%*) was calculated by Equation (4) [43]:

(4) $$Gf\%=\frac{W_{f}}{W_{0}}\times 100$$

where *W_0_* is the initial dry weight of the sample and *W_f_* is the final dry weight, which is the insoluble hydrogel. No HAp was observed by visual and optical microscopy inspections in the solution after an incubation with the dried samples; therefore, we assumed the possible release of solely SA and Coll.

To assess the released components, the concentration of collagen peptides was quantified in both the solution collected after the crosslinking step and in the solution in which the samples were immersed for 24 h for the test. BCA was employed for collagen peptide quantification, with pristine Coll_f_ and Coll_b_ serving as internal standards. The kit consists of two solutions to be mixed according to the manufacturer’s instructions to obtain binding with the protein of interest. A 50 µL aliquot of each collected solution (i.e., the CaCl_2_ solution after the crosslinking step and dH_2_O after 24 h of immersion) was added to 200 µL of the working solution in triplicate to minimize pipetting error; standards were prepared following the same procedure. After an incubation at 37 °C for 30 min, the collagen peptides were detected by measuring absorbance at 570 nm with a VICTOR X3 2030 Multilaber Reader spectrophotometer/photometer/luminometer (Perkin Elmer, Waltham, MA, USA).

Collagen peptide concentrations were determined by comparing the absorbance values to a standard curve. Finally, the released quantities were obtained by multiplying the detected concentrations by the volume of the solutions. The percentage of released SA was then estimated by subtracting the mass of released collagen peptides from the total mass of released material.

### 4.9. Swelling Test

A gravimetric test was used to evaluate the swelling behavior of the two inks. Square samples (10 × 10 × 1 mm^3^, *n* = 3) were 3D printed for each Coll-based formulation and crosslinked with 1 mL of a 1% (*w*/*v*) CaCl_2_ solution for 10 min. Afterward, the samples were soaked with dH_2_O, gently blotted with a paper towel to remove the excess solution, and weighed to determine the initial dry weight (*W_i_*). The samples were placed in a 24-well plate, immersed in 1 mL of DPBS, and incubated at 37 °C for 48 h. At time intervals of 1 h, 2 h, 4 h, 24 h, and 48 h, the samples were gently blotted and weighed to determine the swollen weight (*W_s_*). The swelling percentage (*Sw%*) was calculated using Equation (5) [41]:

(5) $$Sw\%=\frac{W_{s}-W_{i}}{W_{i}}\times 100$$

where *W_i_* is the initial dry weight and *W_s_* is the swollen weight at each time point.

### 4.10. Cytocompatibility Assessment

HDFs, which were isolated and characterized as reported elsewhere [44], were used to perform the in vitro indirect cytotoxicity test on the Coll-based 3D-printed samples. Cells were thawed and suspended in culture medium consisting of DMEM supplemented with 10% fetal bovine serum, 1% PEN-STREP (i.e., 10,000 penicillin units and 10 mg streptomycin in 1 mL of saline solution), 1% L-glutamine (200 mM), and 1% Diflucan. Cell cultures were carried out in an incubator under standard conditions, namely, 37 °C, 95% relative humidity, and a 5% CO_2_/95% air environment. Fresh culture medium was added every 2–3 days. Cells were detached with trypsin EDTA, centrifuged for 5 min at 1200 revolutions per minute (rpm), and counted. Afterwards, the cells were plated in a 24-well plate at a density of 20,000 cells per well and cultured under standard culture conditions until reaching 90% confluence. Samples (*n* = 3) were prepared to have a uniform weight of 0.065 ± 0.01 each, placed in a 24-well plate, and sterilized by UV light exposure for 1 h on each side under the biological hood under sterile conditions. Afterward, 1 mL of complete culture medium was added to each sample, followed by an incubation at 37 °C for 24 h. HDFs were then incubated for 24 h with DMEM conditioned with the samples (*n* = 3).

At the endpoint, cellular metabolic activity was evaluated using resazurin dye. The data were acquired according to manufacturer’s instructions and were expressed as percentages of reduced dye. The system incorporates a specific oxidation–reduction (REDOX) indicator, which undergoes a color change in response to chemical reduction associated with cellular metabolism, and thus it indicates cell viability. This reduction process causes a shift in the REDOX indicator from its oxidized state (blue, i.e., resazurin) to its reduced state (pink, i.e., resorufin). The cytotoxicity protocol was applied, where results were calculated relative to the growth of the positive controls using the same cell density as the samples, which were obtained by adding the dye solution directly to the cells.

Briefly, samples and untreated HDFs as positive controls for growth were incubated for 3 h at 37 °C with the dye diluted in culture medium according to the manufacturer’s instructions. Then, 100 μL of supernatant taken from the sample or control was loaded in 96-well plates. The supernatants were analyzed using a spectrophotometer (Victor 3, PerkinElmer, Waltham, MA, USA) with a double wavelength reading at 570 nm and 600 nm. Finally, the reduced percentage of the dye (*D_red_%*) was calculated by correlating absorbance values and the molar extinction coefficients of the dye at the selected wavelengths. Equation (6) was applied, as follows:

(6) $$D_{red}\%=100\times \frac{\left(\varepsilon_{ox}\right)\lambda_{2}{A\lambda }_{1}-\left(\varepsilon_{ox}\right)\lambda_{1}{A\lambda }_{2}\;of\;test\;agent\;dilution}{\left(\varepsilon_{ox}\right)\lambda_{2}{A{}^{\circ}\lambda }_{1}-\left(\varepsilon_{ox}\right)\lambda_{1}{A{}^{\circ}\lambda }_{2}\;of\;untreated\;positive\;growth\;control}$$

where *ε_ox_* is the molar extinction coefficient of dye oxidized form, *λ_1_* = 570 nm, *λ_2_* = 600 nm, *A* is the absorbance of test wells, and *A°* is the absorbance of cells growing in the positive control well.

### 4.11. Statistical Analysis

Statistical analyses were carried out to assess the significance of the differences observed in the performed tests between HAp/SA/Coll_f_ and HAp/SA/Coll_b_ inks. An independent *t*-test analysis was performed with the Jamovi Software (v. 2.2.5), taking into account of the numerosity of the samples and setting a significance threshold (*p*) equal to 0.05. All data are presented as the means ± standard deviations.

## Figures and Tables

**Table 1:** Locations of the FTIR absorption bands of the collagen samples, together with their assignments to specific vibration modes, namely, stretching (ν) and in plane bending (δ), of the different molecular bonds.

Wavenumber [cm^−1^]	Bond Vibration
861	ν(C-O) ν(C-H) δ(C-O-H) δ(C-O-C)
921	ν(C-O) ν(C-H) δ(C-O-H) δ(C-O-C)
965	ν(C-O) ν(C-H) δ(C-O-H) δ(C-O-C)
1021	ν(C-O)
1077	ν(C-O)
1200	δ(N-H) ν(C-N)
1234	δ(N-H) ν(C-N)
1319	δ(CH_2_) δ(N-H) ν(C-N)
1387	δ(CH_2_) δ(CH_3_)
1442	δ(CH_2_) δ(CH_3_)
1517	δ(N-H) ν(C-N)
1629	ν(C=O)
2870	ν(C-H) ν(CH_3_)
2933	ν(C-H) ν(CH_2_) ν(O-H)
3056	ν(C-H) ν(N-H) ν(O-H)
3270	ν(O-H)
3322	ν(N-H)

**Table 2:** Printability assessment parameters for the HAp/SA/Coll_f_ and HAp/SA/Coll_b_ inks. The values are reported as the means ± standard deviations (*P_r_*: printability; *D_r_*: diffusion rate; *S*: shrinkage).

Ink	*P_r_*	*D_r_*	*S* [%]
HAp/SA/Coll_f_	0.82 ± 0.01	39.51 ± 3.41	13.62 ± 1.41
HAp/SA/Coll_b_	0.85 ± 0.01	43.72 ± 3.39	12.88 ± 0.85

**Table 3:** Gel fraction characterization of the HAp/SA/Coll_f_ and HAp/SA/Coll_b_ inks. The values are reported as the means ± standard deviations.

Ink	Gel Fraction [%]	Collagen Peptides Relased after 24 h [w%]	Crosslinked Alginate [%]
HAp/SA/Coll_f_	88.2 ± 10.1	17.1± 5.2	67.9 ± 29.2
HAp/SA/Coll_b_	90.7 ± 6.7	23.8 ± 6.4	91.4 ± 11.0

**Table 4:** Optimal printing parameters for printing 20 × 20 × 0.6 mm square grids with HAp/SA/Coll_f_ and HAp/SA/Coll_b_ inks exploiting an extrusion-based 3D printing approach (T_h_: printhead temperature; T_b_: print bed temperature).

Ink	Nozzle Gauge	Extrusion Pressure [kPa]	Deposition Speed [mm/s]	z-Offset [mm]	T_h_ [°C]	T_b_ [°C]
HAp/SA/Coll_f_	27G	12–15	5	0.1	RT	20
HAp/SA/Coll_b_	27G	13–16	5	0.1	RT	20

## Data Availability

Data are available upon request to the corresponding authors.
